# Are Text Messages a Feasible and Acceptable Way to Reach Female Entertainment Workers in Cambodia with Health Messages? A Cross-Sectional Phone Survey

**DOI:** 10.2196/mhealth.5297

**Published:** 2016-05-20

**Authors:** Carinne Brody, Sukhmani Dhaliwal, Sovannary Tuot, Michael Johnson, Khuondyla Pal, Siyan Yi

**Affiliations:** ^1^ Touro University California Public Health Program Center for Global Health Research Vallejo, CA United States; ^2^ Touro University California Public Health Program Vallejo, CA United States; ^3^ KHANA Center for Population Health Research Phnom Penh Cambodia

**Keywords:** mHealth, short message service, Cambodia, female sex workers, HIV

## Abstract

**Background:**

Despite great achievements in reducing the prevalence of HIV, eliminating new HIV infections remains a challenge in Cambodia. Entertainment venues such as restaurants, karaoke bars, beer gardens, cafes, pubs, and massage parlors are now considered important venues for HIV prevention efforts and other health outreach interventions.

**Objective:**

The purpose of this study was to explore phone use and texting practices of female entertainment workers (FEWs) in order to determine if text messaging is a feasible and acceptable way to link FEWs to health services.

**Methods:**

This cross-sectional phone survey was conducted in May 2015 with 97 FEWs aged 18–35 years and currently working at an entertainment venue in Phnom Penh.

**Results:**

Of the 96 respondents, 51% reported sending text messages daily; of them, 47% used Khmer script and 45% used Romanized Khmer. Younger FEWs were more likely to report daily texting (*P*<.001). Most FEWs (98%) in this study reported feeling comfortable receiving private health messages despite the fact that 39% were sharing their phone with others. Younger FEWs were less likely to share their phone with others (*P*=.02). Of all of the FEWs, 47% reported owning a smartphone, and younger women were more likely to own a smartphone than were older women (*P*=.08).

**Conclusions:**

The findings from this study support the development of mHealth interventions targeting high-risk groups in urban areas of Cambodia. Our data suggest that mHealth interventions using texting may be a feasible way of reaching FEWs in Phnom Penh.

## Introduction

Despite great achievements in reducing the prevalence of HIV, eliminating new HIV infections remains a challenge in Cambodia. Cambodia is one of the few countries in the world that have reversed their HIV epidemic from generalized to concentrated; it is now confined mainly to individuals who engage in high-risk behaviors such as sex workers [1]. In 2013, it was estimated that the HIV prevalence among the general adult population was 0.6%, reflecting a significant decline from the peak of 2.0% in 1998 [2]. This success was widely attributed to the “100% condom use” program targeting brothel-based commercial relationship, which led to a significant increase in condom use [3-6]. The passage and implementation of the “brothel ban” in 2008, an act that criminalized brothel-based sex work, may be making the situation more complicated because the sex trade has since gone underground, and more women have moved into indirect sex work through the entertainment industry, which is less stigmatized [7]. Entertainment venues include restaurants, karaoke bars, beer gardens, cafes, pubs, and massage parlors [8,9].

In Cambodia, as in many parts of Asia, a common pathway for young women from rural families is to migrate to urban areas to earn a better wage and send money back to their families [9]. Many young women migrate to the capital city to work in garment factories, which are the backbone of Cambodia's economy and employ more than 650,000 females [10], who typically begin working in the factories as teens [11]. These women and girls receive low pay, work long hours, and often struggle to navigate through the new social norms away from family oversight [12,13]. Owing to the poor wages, many seek to supplement or change jobs and move on to more lucrative jobs at entertainment venues. In these roles, many women begin engaging in one or more romantic relationships, which can involve direct or indirect transactional sex [14,15]. Therefore, entertainment venues are an important venue for HIV prevention efforts and other health outreach interventions.

Text messages (short messaging service, SMS) containing health service information and content advising health behavior change have the potential to be an inexpensive, discreet, adaptable, sustainable, and scalable way of reaching the vulnerable populations. Information about service locations and availability, live peer texting, and behavior change messages are some of the ways in which text messages can be used to increase use of critical services such as HIV testing.

Cambodia is the first country in the world in which the number of mobile phone users has surpassed the number using fixed line phones [16]. The number of mobile subscribers in Cambodia reached 20 million at the end of 2013, surpassing the country's population by about 5 million [17]. Mobile phone use by entertainment workers has increased at a similar rate and is now widespread among this population [18]. Worldwide, mobile phones are being used in developing countries to increase contraceptive use [19], improve pharmacovigilance [20], encourage diabetes self-management [21], collect health data [22], increase health knowledge [23], and increase adherence to treatment [24,25]. However, few mobile health (mHealth) interventions have been rigorously evaluated [23,26]. So far, there is rigorous evidence that mobile phone messages can be successfully used to support preventative health care [26-29]. Results from recent studies show that mHealth tools can also be successfully implemented in Cambodia in an urban setting [20], for HIV prevention [30] among young people, using participatory approaches [31-37].

Mobile health is still an emerging field, and new projects, particularly those in developing countries, face challenges. In Cambodia, we have identified a number of challenges for testing mHealth interventions. In terms of technical limitations, mobile users often own multiple subscriber identity module (SIM) cards in order to get cheaper in-network rates and better reception from the competitive phone networks in Cambodia, who also offer deals that entice users to use their SIM cards for a limited period of time [38]. Sharing phones with family members or neighbors, privacy concerns, and varying levels of literacy are additional limiting factors [39]. Furthermore, there is the added concern that most phones in Cambodia lack the ability to text in Khmer script, although the younger generation of tech-savvy Cambodians is more familiar with using a Romanized Khmer language for texting and social media.

The purpose of this study was to explore phone use and SMS practices in order to determine whether text messages are a feasible and acceptable way of linking female entertainment workers (FEWs) to health services in Cambodia.

## Methods

The KHANA Center for Population Health Research reviewed and approved this study on May 15, 2015. The Institutional Review Board Committee of Touro University California approved the study on May 19, 2015 (IRB Application # PH-9015). All participants were informed of the study procedures and purpose and gave their verbal informed consent before participation.

This cross-sectional phone survey was conducted in May 2015. To be eligible for the structured survey, participants needed to be 18-35 years old, female, a mobile phone owner, and currently working at an entertainment venue in Phnom Penh, Cambodia. Three screening questions were used to determine eligibility: “what is your age,” “do you currently work in the entertainment industry in Phnom Penh,” and “do you currently own a mobile phone?”

A list of all FEWs living in Phnom Penh associated with KHANA, the largest national organization providing integrated HIV prevention, care, and support services in Cambodia, was generated by outreach workers working for KHANA’s implementing partners. There were 135 women on the list. One hundred participants were randomly selected from the complete list of FEWs. If a participant did not meet the eligibility criteria or a phone number was no longer in use, another participant was randomly selected from the list. When the list was exhausted, we had managed to recruit 96 participants who were able to be interviewed.

Participants were recruited over the phone using a recruitment script that included screening questions. If they agreed to participate, they were given more information about the study, and their verbal informed consent to participate was required. Once they had given their consent, a structured interview was conducted over the phone. A structured closed-ended questionnaire was developed. The questionnaire covered demographics, text messaging practices, mobile phone use, and privacy concerns. The questionnaire was originally developed in English and translated into Khmer, the national language of Cambodia. The hard copy document was converted into a Google Form to facilitate data input, which was done by multiple research assistants.

Descriptive analyses were conducted to describe participants’ age, type of entertainment venue, and history of garment factory work using n (%) for categorical variables and mean (SD) for continuous variables. The chi-square test or Fisher exact test (when sample sizes were smaller than 5 in 1 cell) was used for categorical variables, and the Student *t* test was used for continuous variables to compare demographic characteristics, SMS use, phone use practices, and attitudes toward privacy and SMS between age groups (≤27 years vs. >27 years). STATA version 13 (StataCorp LP, Texas, USA) was used for all data analyses.

## Results

A total of 96 FEWs participated in this study. Table 1 summarizes the demographic data. The mean age of participants was 27.3 years (SD 5.09). Half of the sample was over 27 years of age. Women worked as beer promoters (39%), restaurant hostesses (16%), karaoke girls (15%), sex entertainment workers (ie, in strip clubs, 15%), and masseuses (9%), as well as in other venues (7%). In total, 35% of participants had worked in a garment factory at some point in the past.

**Table 1:** Demographic data of study participants by age group (n=96).

Demographic variables		Total n (%) (n=96)	Younger FEWs ^a^ n (%) (≤27 years) (n=48)	Oder FEWs n (%) (>27 years) (n=47)	*P*
Age		27.33 (5)	23.0 (3)	31.7 (3)	
	>27 Years	48 (50)			
Type of entertainment work					.04
	Beer promoter	37 (39)	13 (27)	24 (50)	
	Restaurant hostess	15 (16)	10 (21)	5 (10)	
	Karaoke girl	14 (15)	8 (17)	6 (13)	
	Sex entertainment worker	14 (15)	11 (23)	3 (6)	
	Masseuse	9 (9)	2 (4)	7 (15)	
	Other	7 (7)	4 (8)	3 (6)	
Had worked in a garment factory		23 (35)	10 (35)	13 (36)	.96

^a^FEW: female entertainment worker.

Table 2 summarizes data on SMS use. When asked whether they had ever sent a text message, 53% said that they had. Of those, 69% reported sending more than 1 message per day, 22% reported sending about 1 per day, and 10% sent less than 1 per day. When asked what language they used most often when sending text messages, 47% reported using Khmer script, 45% reported using Romanized Khmer, and 8% reported using English.

**Table 2:** Use of short message service by study participants by age group (n=96).

Short message service variables		Total n (%) (n=96)	Younger FEWs^a^ n (%) (≤27 years) (n=48)	Older FEWs n (%) (>27 years) (n=47)	*P*
Have you ever sent a text message on a mobile phone?		51 (53)	37 (77)	14 (29)	<.001
How often do you currently send text messages?					.32
	About 1 per day	11 (21)	6 (16)	5 (36)	
	More than 1 per day	35 (69)	27 (73)	8 (57)	
	Less than 1 per day	5 (10)	4 (11)	1 (7)	
What language do you use most often to send text messages using a mobile phone?					.21
	English	4 (8)	4 (11)	0 (0)	
	Khmer	24 (47)	15 (41)	9 (64)	
	Romanized Khmer	23 (45)	18 (49)	5 (36)	

^a^ FEW: female entertainment worker.

Table 3 summarizes participants’ mobile phone use practices. Of all respondents, 77% owned at least 1 mobile phone, 21% owned 2 mobile phones, and 2% owned 3 mobile phones. When asked about SIM card use, 62% reported currently using 1 SIM card, 37% used 2, and 2% used 3. When asked about the phone that they used most often, 53% of respondents reported using a regular mobile phone and 47% reported using a smartphone.

**Table 3:** Mobile phone use of study participants by age group (n=96).

Mobile phone use variables		Total n (%) (n=96)	Younger FEWs^a^ n (%) (27 and under) (n=48)	Older FEWs n (%) (Over 27) (n=47)	*P*
How many mobile phones do you own right now?					.32
	1	74 (77.1)	34 (70.8)	40 (83.3)	
	2	20 (20.8)	13 (27.1)	7 (14.6)	
	3	2 (2.1)	1 (2.1)	1 (2.1)	
How many SIM cards do you use right now?					.98
	1	59 (61.5)	30 (62.5)	29 (60.4)	
	2	35 (36.5)	17 (35.4)	18 (37.5)	
	3	2 (2.1)	1 (2.1)	1 (2.1)	
When thinking of the mobile phone you use most often, what type is it?					.08
	Regular	50 (52.6)	21 (43.8)	29 (61.7)	
	Smart	45 (47.4)	27 (56.3)	18 (38.3)	

^a^FEW: female entertainment worker, SIM: subscriber identity module.

Table 4 presents data on privacy and mobile phone use. When asked to think about the phone they used most often, 39% reported that they often shared their phone; these FEWs most often shared the phone with work colleagues (43%); family (24%); husbands, boyfriends, or partners (22%); and friends (11%). When asked how comfortable they felt receiving text messages with private health information on their phones, 97% said that they felt comfortable. When asked how likely they were to respond to various types of private health questions, 79% were very likely to respond to a question about eating vegetables, 76% were very likely to respond to a question about smoking, 73% were very likely to respond to questions about condom use, and 87% were very likely to respond to questions about HIV.

**Table 4:** Privacy and short messaging service of study participants by age group (n=96)

Privacy and short messaging service variables		Total (n=96)	Younger FEWs^a^ (27 and under) (n=48)	Older FEWs (Over 27) (n=47)	*P*
Thinking about the phone you use most often, do you share the phone with anyone else?		37 (39)	13 (27)	24 (50)	.02
Who do you share the phone with most often?					.78
	Work colleague	16 (43)	6 (46)	10 (42)	
	Family	9 (24)	2 (15)	7 (29)	
	Husband, boyfriend, or partner	8 (22)	3 (23)	5 (21)	
	Friends	4 (11)	2 (15)	2 (8)	
How comfortable do you feel receiving text messages with private health messages on the phone you most often use?					.56
	Comfortable	93 (97)	46 (96)	47 (98)	
	Not comfortable	3 (3)	2 (4)	1 (2)	
How likely are you to respond to health questions about vegetables?					.16
	Very likely	68 (71)	38 (79)	30 (63)	
	Somewhat likely	12 (13)	6 (13)	6 (13)	
	Not at all likely	6 (6)	2 (4)	4 (8)	
	Do not know	10 (10)	2 (4)	8 (17)	
How likely are you to respond to health questions about smoking?					.49
	Very likely	73 (76)	38 (79)	35 (73)	
	Somewhat likely	7 (7)	2 (4)	5 (10)	
	Not at all likely	16 (17)	8 (17)	8 (17)	
How likely are you to respond to health questions about condom use?					.14
	Very likely	70 (73)	31 (65)	39 (81)	
	Somewhat likely	5 (5)	2 (4)	3 (6)	
	Not at all likely	15 (16)	10 (21)	5 (10)	
	Do not know	6 (6)	5 (10)	1 (2)	
How likely are you to respond to health questions about HIV?					.10
	Very likely	83 (87)	38 (79)	45 (94)	
	Somewhat likely	3 (3)	3 (6)	0 (0)	
	Not at all likely	6 (6)	5 (10)	1 (2)	
	Do not know	4 (4)	2 (4)	2 (4)	

^a^FEW: female entertainment worker.

Younger FEWs were significantly more likely to work at sex entertainment venues and karaoke bars (*P=*.035) and to have ever sent a text message (*P*<.001); however, they were significantly less likely to share their phones with others (*P*=.021). Although not statistically significant at the *P*<.05 level, a greater number of younger FEWs owned smartphones than did older FEWs (*P*=.08).

## Discussion

Our data suggest that mHealth interventions relying on texting may be a feasible way of reaching FEWs in Phnom Penh with health communication programming that aims to improve sexual and reproductive health literacy and access to prevention and care. Half of our respondents sent text messages on a daily basis, and younger FEWs were more likely to report daily texting (*P<*.001). Of those who sent text messages, 47% used Khmer script and 45% used Romanized Khmer. Most FEWs in this study reported feeling comfortable receiving private health messages, despite the fact that around half reported sharing their phone with work colleagues. Younger FEWs were less likely to share their phone with others. Smartphone use was surprisingly high, at 47%, and younger FEWs were more likely to own a smartphone as compared with older women.

The FEWs in our study had higher rates of smartphone ownership and texting in both Khmer script and Romanized Khmer than did those in a nationally representative study. These findings are supported by national data from a recent study on the use of mobile phones. Specifically, in this past study, conducted in 2014, which included a nationally representative sample of 2,066 Cambodians, 93% of respondents reported owning a mobile phone and 28% owned a smartphone, which was a 30% increase from 2013. Additionally, 68% of users knew how to send messages in Khmer script, which represents a 21% increase from 2013, while a quarter (26%) of the sample were able to send messages in Romanized Khmer [40].

These findings may inform future mHealth program designs. Given that more than half of the FEWs in this study did not have smartphones and that this proportion among older women was even less, app-based interventions may not reach an important and influential portion of the population. The delivery of information about where to find services, encouragement on how to protect oneself against HIV, and information on how to make contact with a peer counselor or call for a community-based finger-prick HIV test can all be done using simple text messages. However, an important limiting factor regarding the use of text messages is the low literacy levels in Cambodia, in both Romanized Khmer and Khmer script.

Smartphone use is predicted to increase further over the next decade. In a recent report by Ericsson, a mobile Internet company, usage trends suggest that smartphone subscriptions in Southeast Asia are set to grow approximately five-fold by 2019 [41]. Given the likely increase in smartphone use, the findings from this study suggest that smartphone apps may also be a powerful health tool in addition to text-based interventions.

The limitations of this study include the following. First, the small sample size requires us to be cautious in interpreting our results because of the limited ability to detect statistical significance. Second, we only included FEWs in Phnom Penh who have had some interaction with KHANA in our sample. The levels of mobile phone use and texting frequency reported in this study may therefore represent a more modern view than in other areas of Cambodia. Future studies should include a wider range of the national population, particularly those who have not yet been reached by the KHANA intervention programs.

Although this study had a small sample size, it provides important evidence for the mobile phone use patterns of a specific high-risk population within the context of rapidly increasing rates of mobile phone use in Cambodia. The findings from this study support the development of mHealth interventions targeting high-risk groups in urban areas of Cambodia.
